# Systematically probing the bottom-up synthesis of AuPAMAM conjugates for enhanced transfection efficiency

**DOI:** 10.1186/s12951-016-0178-9

**Published:** 2016-03-31

**Authors:** Elizabeth R. Figueroa, J. Stephen Yan, Nicolette K. Chamberlain-Simon, Adam Y. Lin, Aaron E. Foster, Rebekah A. Drezek

**Affiliations:** Department of Bioengineering, Rice University, 6500 Main St, MS 142, Houston, 77030 TX USA; Bellicum Pharmaceuticals, 2130 W Holcombe Blvd #850, Houston, 77030 TX USA

**Keywords:** Gene therapy, Nanoparticle, Nanotechnology

## Abstract

**Background:**

Gold nanoparticles (AuNPs) have shown great promise as scaffolds for gene therapy vectors due to their attractive physiochemical properties which include biocompatibility, ease of functionalization via the nearly covalent gold-sulfur dative bond, and surface plasmon optical properties. Previously, we synthesized stable AuNP-polyamidoamine (AuPAMAM) conjugates and showed their success in vitro as non-viral gene delivery vectors.

**Results:**

In this study, we systematically perturbed each component of the AuPAMAM conjugates and analyzed the resulting effect on transfection efficiency. Due to the modular, bottom-up nature of the AuPAMAM synthesis, we were able to probe each step of the fabrication process. The relationship between each conjugation parameter and the function of the final vector were investigated. More than fourfold enhanced transfection efficiency was achieved by modifying the PAMAM concentration, PAMAM core chemistry, PAMAM terminus chemistry, and self-assembled monolayer composition of the AuPAMAM conjugates.

**Conclusions:**

This work suggest that AuPAMAM synthesis platform is a promising non-viral gene therapy approach and highlights the importance of inspecting the role of each individual constituent in all nanotechnology hybrid materials.

**Electronic supplementary material:**

The online version of this article (doi:10.1186/s12951-016-0178-9) contains supplementary material, which is available to authorized users.

## Background

Gene therapy is a promising treatment with potential for the management of numerous diseases of acquired and innate origin. Viral delivery vectors are successful in delivering therapeutic DNA, but their efficacy is circumvented by immunogenicity and cost [1, 2]. On the other hand, most non-viral vectors face issues of colloidal stability and transfection efficiency [1–3]. However, gold nanoparticles (AuNPs) have emerged as attractive nanocarriers for non-viral gene delivery due to several attractive properties. AuNPs are particularly attractive scaffolds for the development of non-viral vectors due to their ease of synthesis and size tunability—which allows for enhanced cellular interaction and altered biodistribution, their rich surface chemistry—which facilitates the fabrication of unique NP conjugates, and their biocompatibility [4–9]. AuNPs have been demonstrated as promising delivery vehicles in the literature for in vitro and in vivo gene delivery [10–16]. Previously, we developed a novel gold nanoparticle-dendrimer conjugate for gene therapy where gold nanoparticles were conjugated to polyamidoamine (PAMAM) dendrimers in a facile bottom-up covalent fabrication [17]. PAMAM dendrimers are commercially available, cationic, branched polymers in which growth branches from a core molecule. Their physiochemical properties, such as their highly uniform branched structure and their ability to encapsulate guest molecules in their internal cavities, make PAMAM dendrimers well suited for gene delivery applications [18]. Tailoring the surface properties of AuNP cores by coating them with PAMAM dendrimers produces a hybrid material with novel properties that would not exist with either material alone [16, 19]. These novel properties are created by the physical and chemical interactions between the AuNP core and the dendritic shell. For example, PAMAM dendrimers enhance cellular uptake and endosomal escape, while the AuNP core may reduce dendrimer cytotoxicity and allows ease of characterization by virtue of their optical plasmon properties [14, 16, 19–22].

**Fig. 1:**
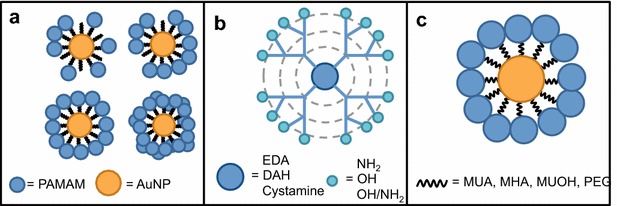
Synthetic parameters investigated. **a** AuPAMAM surface density. **b** PAMAM core and terminal chemistry. **c** SAM composition

The AuPAMAM design is modular, consisting of a 5 nm AuNP core surrounded by an alkanethiol self-assembled monolayer (SAM), and a final layer of generation four PAMAM dendrimers. The stability and efficacy of AuPAMAM particles can be tuned by modifying several parameters: PAMAM surface density, PAMAM core composition, PAMAM terminus composition, and SAM composition. In this work, we systematically investigate the role of each synthetic component of AuPAMAM nanoparticles to generate the optimal vector (Fig. 1). The results and methods presented in this work can be translated to other nanoparticle-cored constructs that utilize a bottom-up synthetic approach. The observations from this work were combined to yield three optimal AuPAMAM configurations for non-viral gene therapy.

## Results and discussion

### AuPAMAM synthesis parameters

**Fig. 2:**
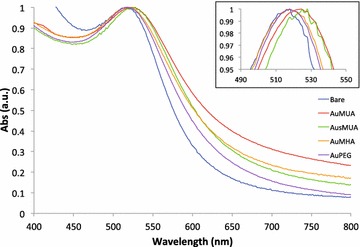
UV-Vis spectra showing peak shifts upon AuSAM formation

The AuPAMAM fabrication method presented here is based on our previously published protocol, with the exception of the PAMAM concentration used [17]. The concentration of PAMAM dendrimer was increased from the original protocol as a greater excess of PAMAM results in fewer PAMAM–PAMAM conjugates and more AuNP-PAMAM conjugates [23]. Briefly, 11-mercaptoundecanoic acid (MUA) was self-assembled onto 5 nm citrate-stabilized AuNPs and salted [24]. Excess MUA was removed by three phosphate buffered saline (PBS) washes in a 10 kDa cutoff centrifuge filter. Once the MUA SAM was formed on the AuNPs, the terminal carboxylic acid moiety of the MUA ligands was activated to bind the aminated dendrimers. To accomplish this, 1-ethyl-3-[3-dimethylaminopropyl]carbodiimide hydrochloride (EDC) and N-hydroxysulfosuccinimide (sulfo-NHS) were added to the AuMUA particles in pH 4.7 MES buffer. EDC, a carbodiimide, catalyzes the formation of amide bonds between the MUA carboxyl and the PAMAM amine groups, while sulfo-NHS stabilizes the coupling reaction via formation of amine reactive esters on the carboxylate. After 15 min, 50-fold molar excess of generation four ethylenediamine (EDA)-cored PAMAM dendrimers were resuspended in PBS, added to the AuMUA solution, and incubated for 2 h. The reaction was quenched by addition of hydroxylamine to cap unbound esters, which proceeded overnight. The next morning, the AuPAMAM particles were concentrated via three water washes in a 50 kDa cutoff centrifuge filter. Following each step of the synthesis process, UV–Visible (UV–Vis) spectroscopy was used to evaluate the stability of the functionalized AuNPs (Fig. 2). Successful conjugation on the surface of AuNPs can be monitored via UV–Vis spectroscopy peak shifts as the refractive index of the AuNP’s surrounding environment changes, resulting in a red shift of the AuNP plasmon peak position. When the MUA SAM was conjugated to the AuNP surface, a 3 nm peak shift from 517 to 520 nm was observed.

The synthesis process outlined above yields the standard AuPAMAM particle referenced throughout this work. The standard AuPAMAM particle synthesis utilizes a 50-fold molar excess of aminated, EDA-cored PAMAM dendrimers and an MUA SAM, and is referred to as MUA-EDA_50_. The variants investigated in this work are shown in Table 1. In each variant, one parameter was altered and compared to the standard particle using UV–Vis spectroscopy, dynamic light scattering (DLS), and cellular viability and transfection in an SK-BR-3 human breast adenocarcinoma cell line under the same optimized transfection conditions.

**Table 1:** AuPAMAM synthetic parameters and nomenclature

AuPAMAM nomenclature	PAMAM molar excess	PAMAM chemistry	SAM composition
Standard	50	EDA core	MUA
Varying PAMAM molar excess			
MUA-EDA_10_	10	EDA core	MUA
MUA-EDA_25_	25	EDA core	MUA
MUA-EDA_100_	100	EDA core	MUA
Varying PAMAM chemistry			
MUA-DAH_50_	50	DAH core	MUA
MUA-Cys_50_	50	Cystamine core	MUA
MUA-OH/NH2_50_	50	1:1 OH:NH_2_	MUA
Varying SAM composition			
MHA-EDA_50_	50	EDA core	MHA
sMUA-EDA_50_	50	EDA core	1:9 MUA:MUOH
PEG-EDA_50_	50	EDA core	PEG
Optimal AuPAMAM vectors			
MHA-DAH_50_	50	DAH core	MHA
PEG-DAH_50_	50	DAH core	PEG
PEG-DAH_100_	100	DAH core	PEG

### Varying PAMAM concentration

First, a titration of PAMAM concentrations was investigated, ranging from 10- to 100-fold molar excess. PAMAM concentration impacts both transfection efficiency and cellular viability, so it is important to quantify the optimal concentration to maximize transfection efficiency without causing significant cell death. Dendrimers are soft nanostructures and thus should be expected to deform and allow denser packing with increasing concentration. In addition to increasing the density of dendrimers on the AuNP surface, increasing the dendrimer concentration may result in electrostatic PAMAM adsorption [25]. Several groups have shown that delivery of free polyethylenimine (PEI) along with PEI/DNA complexes improves transfection efficiency, so it may follow that increasing PAMAM adsorption on the surface of AuPAMAM particles may enhance transfection efficiency as well.

The AuPAMAM vectors were first characterized by UV–Vis spectroscopy (Additional file 1: Figure S1). Decreased spectral broadening and increased peak shift corresponded to increasing PAMAM concentration. Spectral broadening is associated with nanoparticle aggregation, suggesting that at lower PAMAM concentrations, the AuNP surface is not fully passivated by PAMAM dendrimers and is thus susceptible to interparticle aggregation. At lower PAMAM concentrations, multivalent dendrimers may bind to multiple AuMUA particles, resulting in loss of surface charge and aggregation. As the PAMAM concentration is increased, the reaction results in individual AuPAMAM particles with more complete PAMAM surface coverage that are sufficiently charged to electrostatically repel one another and resist aggregation, resulting in more narrow spectra.

**Fig. 3:**
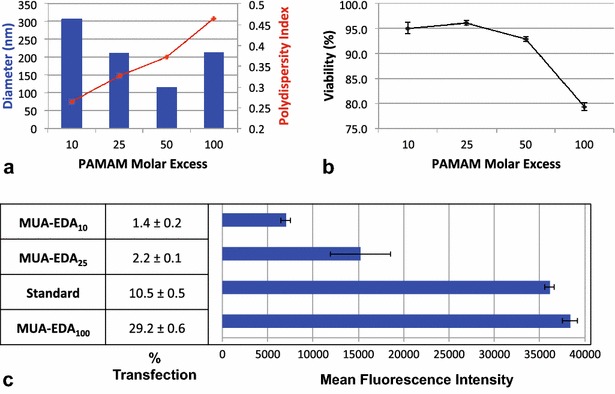
Characterization from varying PAMAM Concentration. **a** AuPAMAM/DNA complex sizes and polydispersity as measured by DLS. **b** Cell viability after complex exposure. **c** Percent transfection and mean fluorescence intensity

Following synthesis, AuPAMAM vectors were mixed with plasmid DNA encoding GFP for 30 min at room temperature, forming electrostatic complexes. Upon complexation with the DNA, the AuPAMAM/DNA complex sizes were measured using DLS, the results of which are shown in Fig. 3. Typical complex sizes for successful gene delivery are around 200 nm [26]. As the PAMAM molar excess increased from 10- to 50-fold, complex sizes decreased from 306 to 116 nm. Upon increasing PAMAM molar excess from 50 to 100, the complex size increased slightly to 214 nm. With increasing PAMAM concentration, the complex polydispersity increased (0.26–0.46), indicating that complex formation becomes less homogeneous at higher PAMAM concentrations.

Next, the AuPAMAM/DNA complexes were introduced to SK-BR-3 human breast adenocarcinoma cells in vitro and transfection was analyzed by measuring the production of GFP by cells via fluorescence microscopy and flow cytometry. As expected, with increasing PAMAM concentration, transfection efficiency improved while viability suffered (Fig. 3). The increase from 50- to 100-fold molar excess of PAMAM yielded the most dramatic change in viability, resulting in a decrease of 13.5 %. However, even at the highest concentration, 100-fold excess of PAMAM, 79.4 % of cells were still viable.

As the PAMAM concentration increases, an increase in percent fluorescence was observed with fluorescence microscopy (Additional file 1: Figure S1) and quantified by flow cytometry. For example, the MUA-EDA_10_ particles resulted in 1.4 % transfection whereas the MUA-EDA_100_ particles transfected nearly 30 % of cells. The mean fluorescence intensity (MFI) also increased with PAMAM concentration, although the two highest concentrations, MUA-EDA_50_ and MUA-EDA_100_, were not significantly different. While the transfection efficiency nearly tripled from MUA-EDA_50_ to MUA-EDA_100_ (10.5 to 29.2 %), the MFI increased only marginally (36,085 to 38,333). This suggests that as the PAMAM molar excess surpasses 50, transfection efficiency per cell remains constant even though the number of transfected cells increases.

One possibility for the observed increase in transfection with higher PAMAM concentrations is electrostatic PAMAM adsorption onto AuSAM particles, since carboxylic acid-functionalized AuNPs have negative zeta potential values [24]. Hu et al. showed with generation seven PAMAM dendrimers that more dendrimers were adsorbed onto an anionic Au surface via electrostatic interaction than when the dendrimers were conjugated in the presence of cross-linking agents EDC and sulfo-NHS. The adsorbed condition also had improved transfection compared to the immobilized condition, purportedly due to improved DNA release from the Au surface into the cells [25]. Overall, the results of this portion of the study indicate that MUA-EDA_50_ and MUA-EDA_100_ are optimal. Depending on the application, MUA-EDA_50_ is ideal in terms of viability whereas MUA-EDA_100_ is optimal in terms of transfection efficiency.

### Varying PAMAM chemistry

**Fig. 4:**
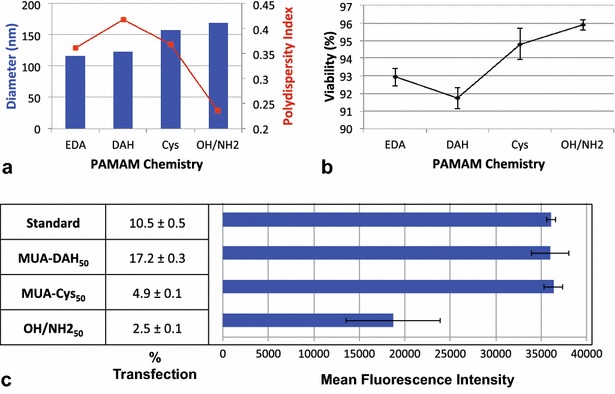
Characterization from varying PAMAM Chemistry. **a** AuPAMAM/DNA complex sizes and polydispersity. **b** Cell viability after complex exposure. **c** Percent transfection and mean fluorescence intensity

Next, the internal structure of the PAMAM dendrimers was modified. The PAMAM core composition is influential in allowing guest molecules, such as water or DNA, to pass through the dendrimer surface groups and enter into the cavities created by branches. This is because the dendrimer core composition, dictated by the core length and the inner branch repulsive forces, affects the asymmetry of the structure. The type and length of core can be optimized to increase encapsulation efficiency, so that the core will push away neighboring layers, resulting in larger cavities around the dendrimer core where guest molecules can reside [18]. We considered EDA, (diaminohexane) DAH, and cystamine cores. EDA-cored, amine-terminated PAMAM constitutes the standard AuPAMAM dendrimer. DAH cores are four carbons longer than EDA cores. Cystamine has a disulfide bond, with two carbons flanking each sulfur (Fig. 4).

For all PAMAM core chemistries investigated, the UV–Vis spectra of the final AuPAMAM particles did not vary significantly (Additional file 2: Figure S2). This is likely because the PAMAM dendrimer diameter provides the most significant change to the refractive index and thus any minor contributions from the dendrimer core are not seen. For all core chemistries evaluated, in vitro cellular viability was 91.7 % or above, indicating that the SAM composition does not significantly increase cytotoxicity. In addition, AuPAMAM/DNA complex sizes ranged narrowly from 116 to 158 nm. Thus, it would appear that changing the core chemistry of the PAMAM dendrimers does not change the AuPAMAM structure dramatically. However, the transfection efficiency varied significantly with PAMAM core chemistry. MUA-Cys_50_ particles performed the worst, transfecting only 4.9 % of cells, no better than PEI (7 %) or PAMAM alone (8.4 %). MUA-DAH_50_ particles performed the most impressively, transfecting 17.2 % of cells, compared to the standard MUA-EDA_50_ particle, which transfected 10.5 % of cells. However, the MFI of the three core chemistries were very similar, ranging from 35,986 to 36,342. This indicates that, while MUA-DAH_50_ particles are transfecting more cells, they are not necessarily transfecting individual cells more efficiently than the standard or MUA-Cys_50_ AuPAMAM particles.

In changing the PAMAM core from EDA to DAH, the flexibility of the PAMAM conformation is improved. Coarse-grained molecular dynamics simulations have shown that DAH-cored PAMAM dendrimers are larger than EDA at pH 5 and 7 due to the increased length of the DAH core, resulting in a structure with more repulsion between the branches and core. The DAH-cored dendrimers also have significantly less back folding of the terminal branches, resulting in more inner cavities and enhanced encapsulation efficiency [18]. We suspect that the enhanced flexibility imparted by the core allows the dendrimers to bind more readily within the DNA grooves, thus improving complexation and transfection efficiency. Kavyani et al. postulate that increasing the number of carbons in the core enhances the aqueous solubility of guest molecules by increasing structural asymmetry and thus creating cavities in the dendrimer structure [18].

In the case of cystamine-cored PAMAM, an increase in transfection efficiency was expected with an increase in flexibility, similar to the results observed for DAH. Surprisingly, MUA-Cys_50_ particles had the lowest transfection efficiency. One hypothesis for this unexpected trend is that upon exposure to the intracellular environment, elevated levels of glutathione reduce the dendrimer’s disulfide bond, resulting in decreased AuPAMAM charge and subsequently decreased DNA affinity due to the loss of the reduced portion of the dendrimer. Several groups have studied the effect of glutathione-mediated release from disulfide moieties and cystamine cored PAMAM dendrimers, supporting this hypothesis [27–29]. In addition, disulfide bonds are chemically rigid and thus the increase in length with respect to EDA may not be sufficient to counteract the increased rigidity imparted by the disulfide bond [30, 31].

The surface charge density of PAMAM also affects its ability to condense DNA; higher surface charge density will result in higher transfection efficiency, but is often more cytotoxic [32]. Cytotoxicity studies have shown that amine-terminated dendrimers interact strongly with cell lipid bilayers, which can produce permanent holes in the cell membrane [33]. Thus, we investigated a hydroxyl-terminated PAMAM terminus and a 1:1 molar mixture of amine-terminated and hydroxyl-terminated (OH/NH_2_) PAMAM surface. The hydroxylated AuPAMAM particles did not result in any transfection and were thus excluded from subsequent results. The MUA-OH/NH2_50_ AuPAMAM particles were significantly less effective than the standard AuPAMAM particle, with only 2.5 % transfection compared to 10.5 % for the standard, and they did not surpass the PEI and PAMAM controls (Additional file 3: Figure S3). In addition, the MFI of the MUA-OH/NH2_50_ particles was significantly lower than the standard (18,725 vs. 36,085). These results agree with other studies investigating the use of hydroxylated PAMAM dendrimers for gene delivery [34]. Thus, in terms of PAMAM chemistry we have found that the DAH-cored dendrimer is superior to EDA and cystamine, and that amine-terminated PAMAM is superior to hydroxylated or mixed termini PAMAM.

### Varying SAM composition

It has been reported that SAM surfaces with reactive functional groups are well suited for covalent immobilization of dendrimers and also stabilize AuNPs by providing steric hindrance against AuNP aggregation [25]. We investigated various SAMs to probe their effect on AuPAMAM stability and transfection efficiency. The SAM composition is particularly sensitive in nanoparticle applications as monolayer structure is dependent on the nanoparticle diameter. With the 5 nm AuNP cores used in this work, there is a relatively large radius of curvature, thus decreasing steric hindrance between thiolated chains as one moves away from the AuNP surface. As a result, there is more conformational freedom in the SAM on a 5 nm core than a larger core, allowing for more guest interactions with DNA due to the hydrocarbon portion of the SAM being more accessible for solvation [35]. A variety of carboxylic acid-terminated molecular adsorbates having various chain lengths of alkane or polyethylene glycol (PEG) moieties were used to functionalize the surface of AuNPs. Carboxy-PEG_12_-thiol (635 Da), 11-MUA, 6-mercaptohexanoic acid (MHA), and a 1:9 molar ratio combination of MUA and 11-mercapto-1-undecanol (MUOH) were explored. The latter is referred to as spaced MUA (sMUA) because the EDC/sulfo-NHS reaction activates only the MUA and not the MUOH ligands, resulting in spaced PAMAM binding sites. Again, the MUA-EDA_50_ AuPAMAM will be considered the standard.

**Fig. 5:**
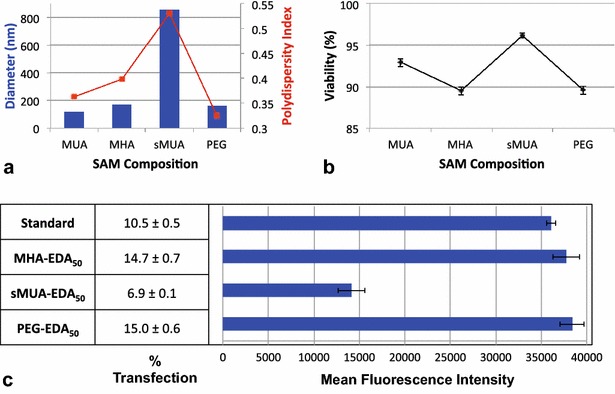
Characterization from varying SAM Composition. **a** AuPAMAM/DNA complex sizes and polydispersity. **b** Cell viability after complex exposure. **c** Percent transfection and mean fluorescence intensity

Following SAM formation on the AuNP surface, the PEG and MHA SAMs showed the smallest red shift, and the MUA and sMUA SAMs exhibited the largest red shift. The MUA and MHA SAMs resulted in broader spectra than the sMUA and PEG SAMs (Fig. 2). Following PAMAM conjugation, the sMUA-EDA_50_ particles exhibited the largest red shift and broadening, likely due to decreased stability from charge-induced aggregation (Additional file 4: Figure S4). This aggregation was also reflected in the sMUA-EDA_50_/DNA complex size, which was nearly 900 nm (Fig. 5). The PEG-EDA_50_ particles had the narrowest spectra, suggesting that they are colloidally stable. Following complexation with DNA, the standard (MUA-EDA_50_), MHA-EDA_50_, and PEG-EDA_50_ complexes were similar in size, ranging from 116 to 165 nm. However, the sMUA-EDA_50_ complexes were much larger, as reported above. Cellular viability was minimally affected by the varying SAM parameters, and ranged from 89.5 to 96.2 %.

After synthesis and characterization, the MHA, MUA and sMUA alkanethiol SAMs were evaluated in in vitro transfection experiments. The shorter chained MHA-EDA_50_ particles were significantly more effective at transfecting cells than the standard MUA-EDA_50_ particles. The MFI of the two ligands is not significantly different, however. Alkanethiol chains with more than 10 carbon atoms, like MUA, result in more highly ordered, dense SAMs than shorter chains, like MHA. A greater packing density is postulated with an increase of the ligand length due to enforced interstrand Van der Waals interactions [36]. Thus, MUA will form a more uniform, ordered, dense SAM than MHA whereas shorter chains like MHA are more likely to possess pinholes, gauche defects, and collapsed-site defects due to imperfect adsorption [24, 37]. As a result, the MHA-EDA_50_ particles are more flexible and accessible to solvation than the standard, resulting in enhanced DNA interactions and transfection. In contrast, the sMUA-EDA_50_ vectors transfected significantly fewer cells than the standard (6.9 vs. 10.5 %, respectively) and with a significantly lower MFI (14,148 vs. 36,085). We hypothesize that at a 50-fold molar excess of PAMAM, the sMUA surface is nearly fully functionalized, but the standard MUA SAM possesses more binding sites and thus is more cationic and capable of condensing DNA.

Next, we transitioned from a hydrophobic alkyl chain SAM to a hydrophilic PEG SAM. The PEG-EDA_50_ vector performs significantly better than the standard in terms of transfection (15 vs. 10.5 %, respectively). As with MHA-EDA_50_, the PEG-EDA_50_ MFI was not significantly different from the standard. The PEG utilized in this experiment is a 12 PEG unit linker that is longer than the MUA ligand, which suggests that increasing the hydrophilicity of the SAM may be beneficial. This would agree with our previous observation that decreasing the length of the alkyl chain enhanced transfection, as this also decreases hydrophobicity.

### Optimization of synthetic parameters

New vectors were synthesized taking into account the optimal parameters: 50- and 100-fold molar excess of DAH-cored, amine-terminated PAMAM dendrimers on a MHA or PEG SAM. In synthesizing these four optimal vectors, the MHA-DAH_100_ combination was not colloidally stable and fell out of solution before the synthesis was complete. Colloidal AuNPs are subject to Brownian motion, resulting in frequent collisions between particles. Attractive van der Waals forces and repulsive electrostatic and steric forces govern the collision, and the balance of these forces determines colloidal stability. We suspect the MHA-DAH_100_ AuPAMAM vectors were not stable due to the fact that the shorter alkyl chain was not sufficient to passivate the Au surface from the high concentration of cationic dendrimers, resulting in inter-particle binding or aggregates. MHA-DAH_50_, PEG-DAH_50_, and PEG-DAH_100_ vectors were all colloidally stable (Additional file 5: Figure S5).

**Fig. 6:**
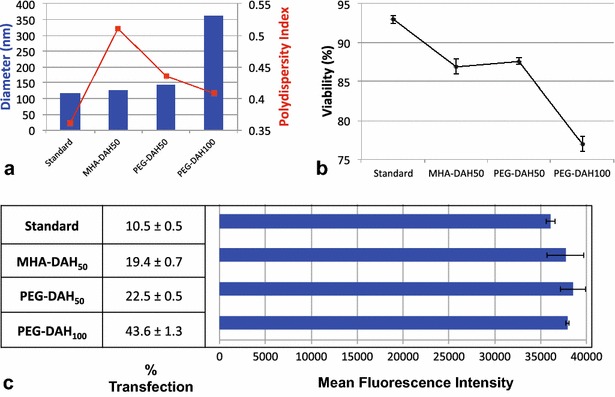
Optimization of synthesis parameters; **a** AuPAMAM/DNA complex sizes and polydispersity. **b** Cell viability after complex exposure. **c** Percent transfection and mean fluorescence intensity

The combination of short alkane SAM with the flexible DAH core yielded a successful vector that transfected 19.4 % of cells—nearly double that of the standard AuPAMAM vector (Fig. 6). The viability of the MHA-DAH_50_ AuPAMAM vector was not significantly affected, at 86.9 %. The PEG-DAH_50_ vector yielded a slightly improved transfection compared to MHA-DAH_50_ at 22.5 %, slightly more than twice the standard AuPAMAM vector. Again, the change in viability was not significantly different (87.6 %). Increasing the molar excess of PAMAM to 100 nearly doubled the transfection efficiency compared to PEG-DAH_50_ vectors. Ultimately, the PEG-DAH_100_ vectors were the most effective, transfecting 43.6 % of cells, an improvement of more than 4× the standard AuPAMAM vector. Cellular viability decreased somewhat to 77 %, indicating that PAMAM molar excess should be modified to suit the viability needs of each application. In each of the optimized cases, the MFI is not significantly improved, indicating that transfection efficiency per cell has not changed significantly.

## Conclusions

Gold nanoparticle-based delivery platforms have proven advantageous in many biomedical applications. When combined with cationic PAMAM dendrimers, a novel class of AuPAMAM nanoparticles emerges as effective non-viral gene therapy vectors. In this work, we have highlighted the benefits of the modular AuPAMAM delivery platform, which enables us to probe specific synthetic parameters and optimize transfection efficiency. We first synthesized AuPAMAM particles, varying one parameter at a time and comparing to the standard AuPAMAM particle, MUA-EDA_50_. The particles were characterized by UV–Vis spectroscopy to observe the spectral shift following each step of the synthesis. All particles were stable, aside from the sMUA SAM particles which aggregated. DLS was utilized to characterize the hydrodynamic diameter and polydispersity of the AuPAMAM/DNA complexes. AuPAMAM/DNA complexes ranged from approximately 100 to 300 nm, with the exception of sMUA-EDA_50_ complexes, which were nearly 900 nm with high polydispersity. We found that with increasing PAMAM concentration, transfection efficiency increased. At the highest PAMAM concentration tested, there was more than fourfold improvement in percent transfection, but also an associated decrease in viability from 92.9 to 79.4 %. Thus, PAMAM molar excess should be chosen with the balance of transfection and viability in mind. The physiochemical PAMAM properties were probed by changing the dendrimer’s core and terminal molecules. Amine-terminated DAH-cored dendrimers were optimal due to enhanced DNA encapsulation and condensation ability. Finally, the SAM composition was evaluated by exploring hydrophobic and hydrophilic ligands of various lengths. Here, we found that the PEG SAM was superior to the MUA, MHA, and sMUA SAMs. Future work should expand upon the impact of SAM composition by exploring other hydrophobic and hydrophilic ligands and PEG of different molecular weights. Our findings suggest that AuPAMAM nanoparticle vectors can be further optimized and the AuPAMAM synthesis platform is a promising non-viral gene therapy approach. This work also highlights the importance of interrogating the role of each synthetic component in all nanotechnology hybrid materials.

## Methods

### Materials

11-MUA, 11-MUOH, 6-MHA, 25 kDa branched PEI, Tween 20, generation four PAMAM dendrimers with EDA core, DAH core, cystamine core or hydroxyl termini were purchased from Sigma Aldrich (St. Lewis, MO). EDC and Sulfo-NHS were purchased from Thermo Scientific (Waltham, MA). 5 nm citrate stabilized colloidal AuNPs were purchased from Ted Pella (Redding, CA). Plasmid DNA with cytomegalovirus (CMV) promoter and enhanced green fluorescent protein (eGFP) as the reporter gene (pCMV-eGFP, 4.7 kb) were obtained from Clark Needham at Rice University [38]. SK-BR-3 cell line, cell culture medium and PBS were purchased from ATCC (Manassas, VA). MES buffered saline and CT(PEG)_12_ were purchased from Pierce (Rockford, IL). Amicon Ultra-15 10 and 50 kDa Centrifuge Concentrators were purchased from Millipore (Billerica, MA). Hydroxylamine was purchased from Alfa Aesar (Ward Hill, MA). Propidium iodide (PI) Staining Solution was purchased from eBiosSciences (San Diego, CA). All other chemicals were purchased from Sigma Aldrich (St. Lewis, MO) or Fisher Scientific (Waltham, MA) unless otherwise stated.

### AuPAMAM synthesis

First, 11-MUA, 6-MHA, CT(PEG)_12_ (PEG), or a 1:9 molar ratio of MUA and 11-MUOH was added to 5 nm AuNPs (5 × 10^13^ particles/ml) to a final concentration of 83.33 μM in 12 ml. After 24 h, the solution was raised to 0.1 M NaCl, 100 mM sodium phosphate, and 0.1 % v/v Tween 20 and incubated for another 24 h. Next, unbound SAM ligand (MUA, MHA, PEG, or MUOH) was removed by centrifuge filtration (10,000 MWCO) at 2500*g* for 20 min and washed three times with PBS. After the last PBS wash, the AuSAM NPs were resuspended in MES buffer. EDC and sulfo-NHS linkers were added to a final concentration of 0.44 and 0.59 mM for 15 min. Then, the particles were added to generation four EDA, DAH, or cystamine-cored PAMAM dendrimers in PBS. To estimate the amount of dendrimer needed for the conjugation, a surface packing model was used [17]. A 50-fold molar excess of the maximal dendrimer binding concentration was used for the standard conjugation process. After 2 h, 1 mL of 50 mM hydroxylamine (pH 7) in PBS was added to the solution and left nutating overnight to backfill any unconjugated sulfo-NHS esters. Lastly, the solution was washed three times using a centrifuge filter (50,000 MWCO) with sterile DNase free deionized (DI) water. The AuPAMAM nanoparticles were resuspended in DI water and stored at 4 °C until further use. The particles were sonicated before use.

### AuPAMAM characterization

All AuNP, AuMUA, and AuPAMAM particles were sonicated and characterized by UV–Vis absorbance spectroscopy in 1 mm path cells using baseline correction in a Cary 60 UV–Vis (Agilent Technologies). The particle size was measured using a 90-Plus Particle Size Analyzer (Brookhaven) by diluting 30 μL of AuPAMAM nanoparticles in 3 mL of DI water. The volume size distribution mean and polydispersity were reported using the AuNP refractive index values, and represent three separate 3-min runs.

### Cell culture

SK-BR-3 cells were cultured in a humidified incubator (5 % CO_2_, 37 °C). The cells were suspended in McCoy’s 5A and supplemented with 10 % Fetal Bovine Serum (FBS) and 1 % Penicillin–Streptomycin. Complete media was used throughout all experiments.

### AuPAMAM/DNA transfection

For transfection assays, 75,000 cells/well were added to 24-well plates and grown overnight. Both AuPAMAM (5.9 × 10^12^ NP/mL) and DNA (0.8 μg) solutions were diluted with ultra pure DI water to a final volume of 50 μL and then mixed together. Water was used as the solvent in order to prevent charge screening effects prior to complex formation. The final volume of the polyplexes per well was 100 μL. The polyplex solutions were vortexed gently and incubated for 20 min at room temperature, then added to cells in a 24-well plate. The next day, wells were rinsed with PBS and complete media was added. At 48 h, medium was changed again and GFP expression of all conditions was visualized using a Zeiss Axio Observer inverted microscope. Transfection efficacy of fixed cells was measured using flow cytometry (BD FACSCanto II). The data presented are the mean fluorescent signals for 10,000 cells.

### Cell fixation

Cells were aspirated, and thawed trypsin (200 μL/well) was added and incubated for 5 min. Next, 800 μL of complete media was added and the contents of each well were transferred to labeled flow tubes and spun at 400 g for 5 min. The tubes were decanted and the cell pellets were resuspended in 1 mL of PBS. The tubes were spun again and decanted. Cells were then resuspended in 300 μL of BD Cytofix and stored on ice until analysis.

### Viability experiments

Following cellular transfection, viability was assessed using PI. Cells were fixed and stored on ice. PI (1 μL) was added to each flow tube 5 min prior to analyzing the samples (BD FACSCanto II). The data presented are the mean fluorescent signals for 10,000 cells. Compensation controls for GFP and PI were acquired prior to experimental acquisition.

### Statistical analysis

All data are expressed as mean ± standard deviation. Statistical differences were evaluated using ANOVA and Tukey’s HSD and considered significant at p < 0.05. All figures shown were obtained from at least three independent experiments. Any images shown are representative of the entire experiment.

## Additional files

10.1186/s12951-016-0178-9 Varying PAMAM concentration. Fluorescence microscopy of GFP expression in SK-BR-3 cells transfected with A) MUA-EDA_10_, B) MUA-EDA_25_, C) MUA-EDA_50_, D) MUA-EDA_100_ vectors. E) UV-visible spectroscopy showing peak shifts after AuPAMAM synthesis.
10.1186/s12951-016-0178-9 Varying PAMAM chemistry. Fluorescence microscopy of GFP expression in SK-BR-3 cells transfected with A) MUA-EDA_50_, B) MUA-DAH_50_, C) MUA-Cys_50_, D) MUA-OH/NH250 vectors. E) UV/visible spectroscopy showing peak shifts after AuPAMAM synthesis.
10.1186/s12951-016-0178-9 Experimental controls. Fluorescence microscopy of GFP expression in SK-BR-3 cells transfected with A) PAMAM amine:phosphate ratio (N:P) = 10, B) PEI N:P = 7.5, C) PEI N:P = 20, D) DNA alone. E) Cell viability after exposure to controls. F) Percent transfection and mean fluorescence intensity of controls.
10.1186/s12951-016-0178-9 Varying SAM Composition. Fluorescence microscopy of GFP expression in SK-BR-3 cells transfected with A) MUA-EDA_50_, B) MHA-EDA_50_, C) sMUA-EDA_50_, D) PEG-EDA_50_ vectors. E) UV/visible spectroscopy showing peak shifts after AuPAMAM synthesis.
10.1186/s12951-016-0178-9 Optimizing Parameters. Fluorescence microscopy of GFP expression in SK-BR-3 cells transfected with A) MUA-EDA_50_, B) MHA-DAH_50_, C) PEG-DAH_50_, D) PEG-DAH_100_ vectors. E) UV/visible spectroscopy showing peak shifts after AuPAMAM synthesis.

## Abbreviations

AuNP: gold nanoparticle
CMV: cytomegalovirus
DAH: diaminohexane
DLS: dynamic light scattering
EDA: ethylenediamine
EDC: 1-ethyl-3-[3-dimethylaminopropyl]carbodiimide hydrochloride
eGFP: enhanced green fluorescent protein
MFI: mean fluorescence intensity
MHA: mercaptohexanoic acid
MUA: mercaptoundecanoic acid
MUOU: mercapto-1-undecanol
PAMAM: polyamidoamine
PEG: polyethylene glycol
PEI: polyethylenimine
PI: propidium iodide
SAM: self-assembled monolayer
sMUA: spaced MUA
Sulfo-NHS: N-hydroxysulfosuccinimide
