# Supplementary material for: Systematically probing the bottom-up synthesis of AuPAMAM conjugates for enhanced transfection efficiency
Source: J Nanobiotechnology. 2016 Mar 31;14:24. doi: 10.1186/s12951-016-0178-9 (PMC4815207; doi:10.1186/s12951-016-0178-9)
Supplement: Supplementary file 1 — 10.1186/s12951-016-0178-9 Varying PAMAM concentration. Fluorescence microscopy of GFP expression in SK-BR-3 cells transfected with A) MUA-EDA10, B) MUA-EDA25, C) MUA-EDA50, D) MUA-EDA100 vectors. E) UV-visible spectroscopy showing peak shifts after AuPAMAM synthesis. [file 12951_2016_178_MOESM1_ESM.pdf]

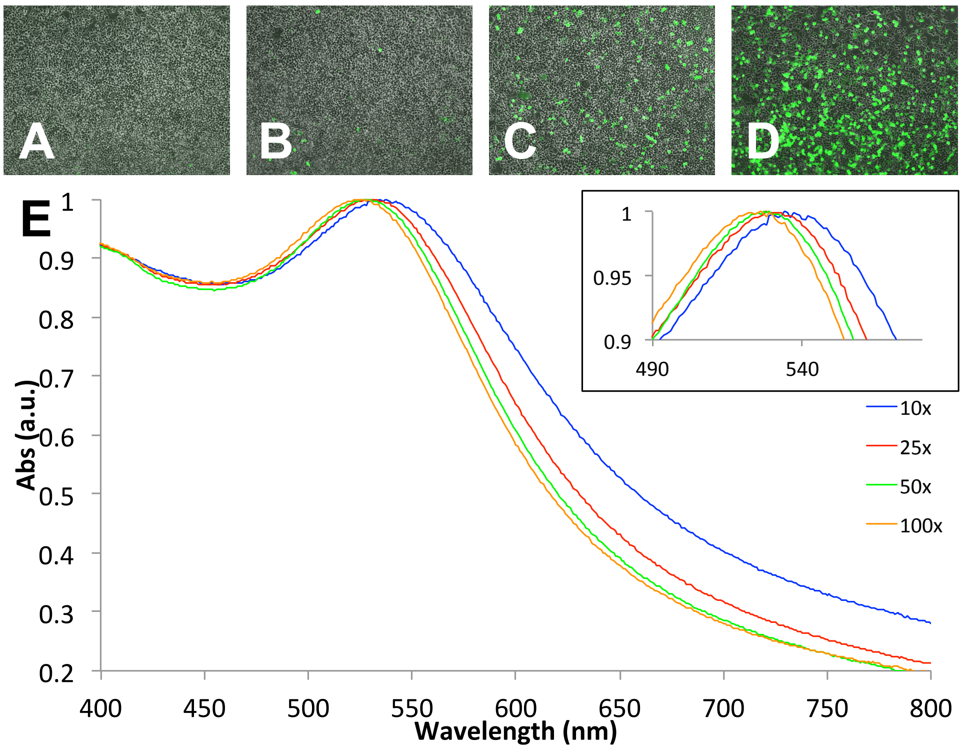

Figure S1. Varying PAMAM Concentration. Fluorescence microscopy of GFP expression in SK-BR-3 cells transfected with A) MUA-EDA_{10}, B) MUA-EDA_{25}, C) MUA-$EDA_{50,}$ D) MUA-EDA_{100} vectors. E) UV-visible spectroscopy showing peak shifts after AuPAMAM synthesis.
