# Supplementary material for: Systematically probing the bottom-up synthesis of AuPAMAM conjugates for enhanced transfection efficiency
Source: J Nanobiotechnology. 2016 Mar 31;14:24. doi: 10.1186/s12951-016-0178-9 (PMC4815207; doi:10.1186/s12951-016-0178-9)
Supplement: Supplementary file 2 — 10.1186/s12951-016-0178-9 Varying PAMAM chemistry. Fluorescence microscopy of GFP expression in SK-BR-3 cells transfected with A) MUA-EDA50, B) MUA-DAH50, C) MUA-Cys50, D) MUA-OH/NH250 vectors. E) UV/visible spectroscopy showing peak shifts after AuPAMAM synthesis. [file 12951_2016_178_MOESM2_ESM.pdf]

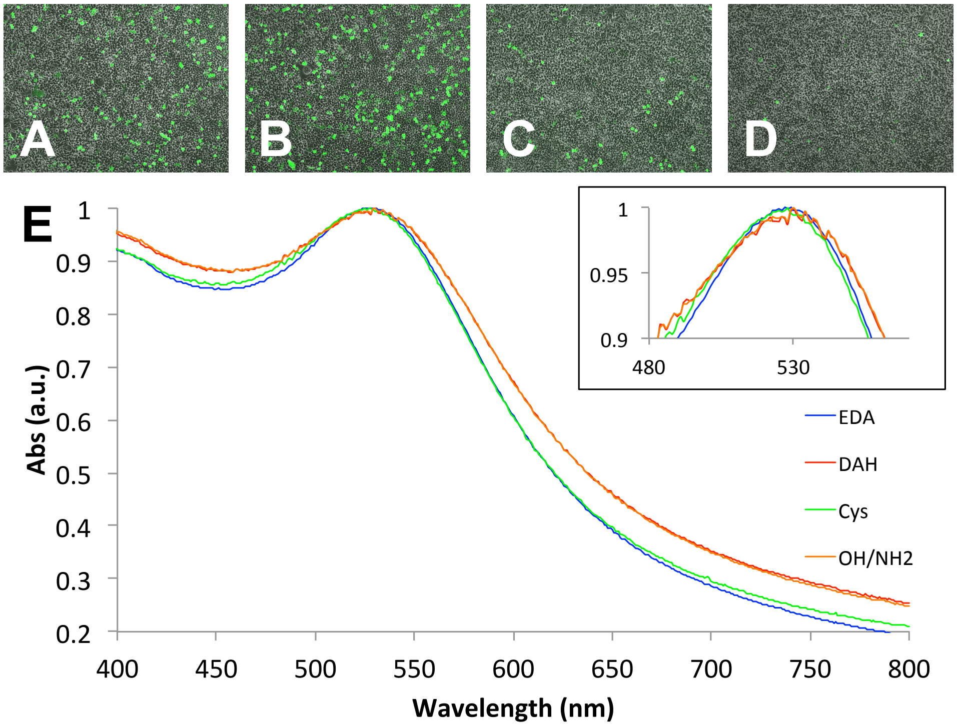

Figure S2. Varying PAMAM Chemistry. Fluorescence microscopy of GFP expression in SK-BR-3 cells transfected with A) MUA-EDA_{50}, B) MUA-DAH_{50}, C) MUA-Cys_{50}, D) MUA-$OH/NH2_{50}$ vectors. E) UV/visible spectroscopy showing peak shifts after AuPAMAM synthesis.
