# Supplementary material for: Systematically probing the bottom-up synthesis of AuPAMAM conjugates for enhanced transfection efficiency
Source: J Nanobiotechnology. 2016 Mar 31;14:24. doi: 10.1186/s12951-016-0178-9 (PMC4815207; doi:10.1186/s12951-016-0178-9)
Supplement: Supplementary file 3 — 10.1186/s12951-016-0178-9 Experimental controls. Fluorescence microscopy of GFP expression in SK-BR-3 cells transfected with A) PAMAM amine:phosphate ratio (N:P) = 10, B) PEI N:P = 7.5, C) PEI N:P = 20, D) DNA alone. E) Cell viability after exposure to controls. F) Percent transfection and mean fluorescence intensity of controls. [file 12951_2016_178_MOESM3_ESM.pdf]

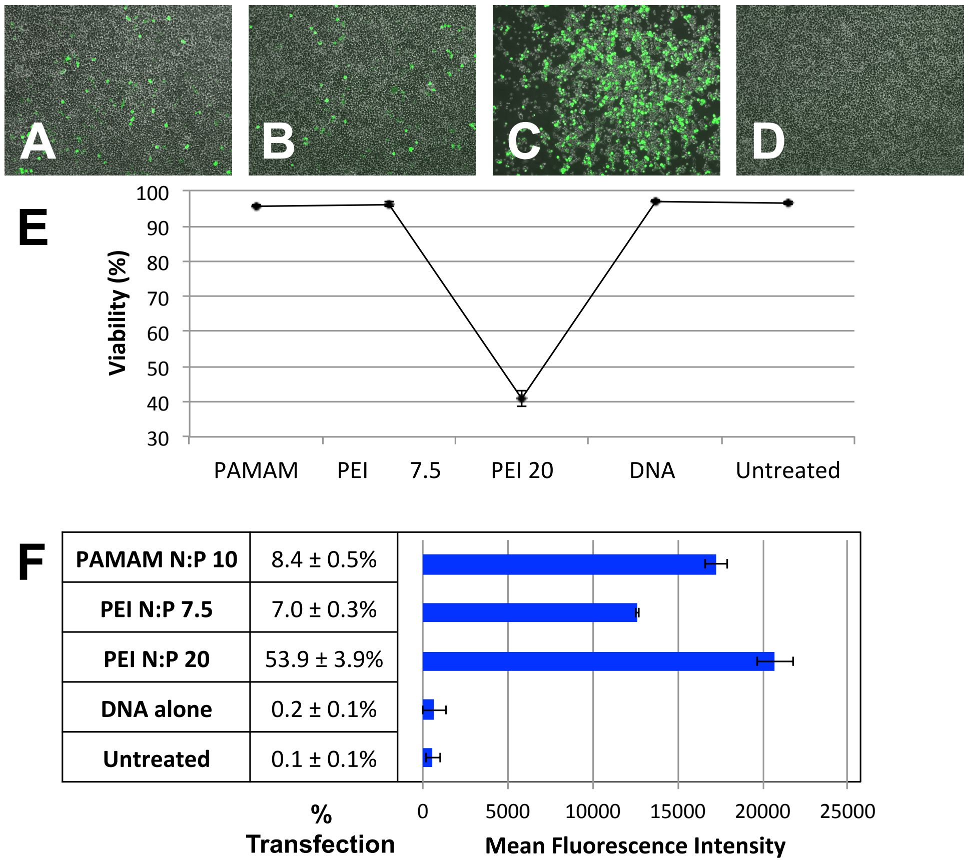

Figure S3. Experimental controls. Fluorescence microscopy of GFP expression in SK-BR-3 cells transfected with A) PAMAM amine:phosphate ratio (N:P) = 10, B) PEI N:P = 7.5, C) PEI N:P = 20, D) DNA alone. E) Cell viability after exposure to controls. F) Percent transfection and mean fluorescence intensity of controls.
