# Supplementary material for: Systematically probing the bottom-up synthesis of AuPAMAM conjugates for enhanced transfection efficiency
Source: J Nanobiotechnology. 2016 Mar 31;14:24. doi: 10.1186/s12951-016-0178-9 (PMC4815207; doi:10.1186/s12951-016-0178-9)
Supplement: Supplementary file 4 — 10.1186/s12951-016-0178-9 Varying SAM Composition. Fluorescence microscopy of GFP expression in SK-BR-3 cells transfected with A) MUA-EDA50, B) MHA-EDA50, C) sMUA-EDA50, D) PEG-EDA50 vectors. E) UV/visible spectroscopy showing peak shifts after AuPAMAM synthesis. [file 12951_2016_178_MOESM4_ESM.pdf]

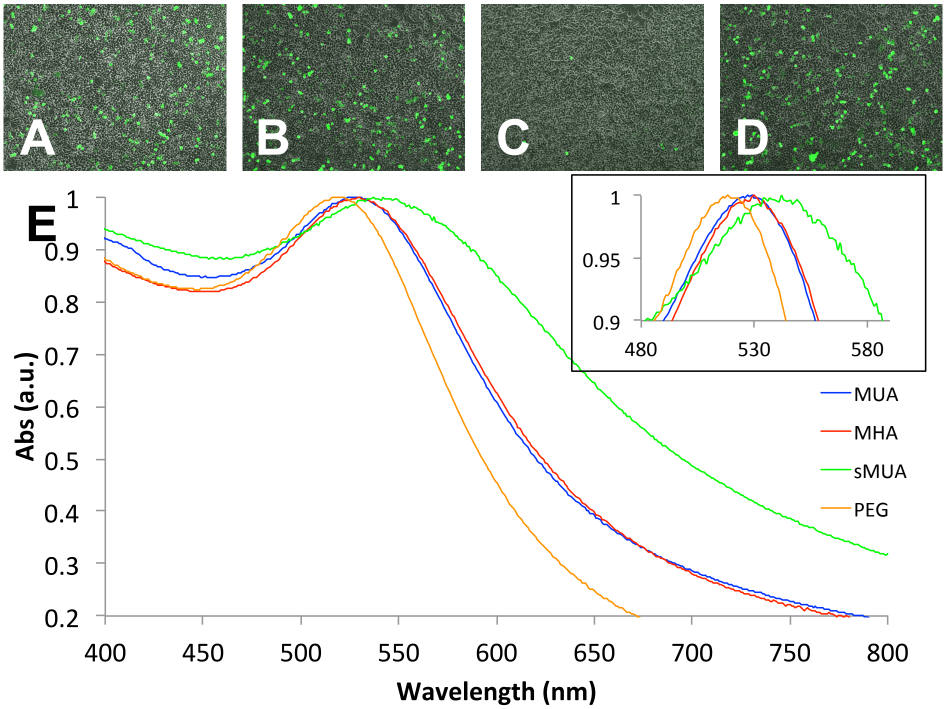

Figure S4. Varying SAM Composition. Fluorescence microscopy of GFP expression in SK-BR-3 cells transfected with A) MUA-EDA_{50}, B) MHA-EDA_{50}, C) sMUA-$EDA_{50,}$ D) PEG-EDA_{50} vectors. E) UV/visible spectroscopy showing peak shifts after AuPAMAM synthesis.
