# Supplementary material for: Systematically probing the bottom-up synthesis of AuPAMAM conjugates for enhanced transfection efficiency
Source: J Nanobiotechnology. 2016 Mar 31;14:24. doi: 10.1186/s12951-016-0178-9 (PMC4815207; doi:10.1186/s12951-016-0178-9)
Supplement: Supplementary file 5 — 10.1186/s12951-016-0178-9 Optimizing Parameters. Fluorescence microscopy of GFP expression in SK-BR-3 cells transfected with A) MUA-EDA50, B) MHA-DAH50, C) PEG-DAH50, D) PEG-DAH100 vectors. E) UV/visible spectroscopy showing peak shifts after AuPAMAM synthesis. [file 12951_2016_178_MOESM5_ESM.pdf]

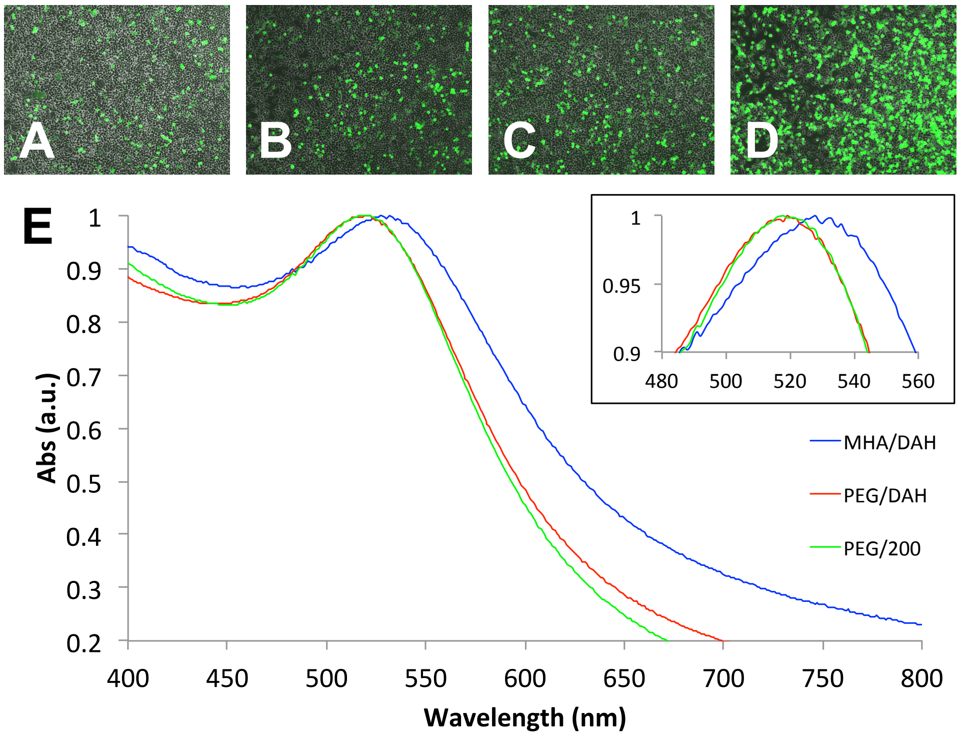

Figure S5. Optimizing Parameters. Fluorescence microscopy of GFP expression in SK-BR-3 cells transfected with A) MUA-EDA$_{50}$, B) MHA-DAH$_{50}$, C) PEG-DAH$_{50,}$ D) PEG-DAH$_{100}$ vectors. E) UV/visible spectroscopy showing peak shifts after AuPAMAM synthesis.
